# Healthcare professionals' perception of equipment and infrastructure as determinants of quality in anesthesia and intensive care units

**DOI:** 10.3389/fmed.2026.1797446

**Published:** 2026-05-26

**Authors:** Gabriel-Petre Gorecki, Dorel Săndesc, Horaţiu Moldovan, Alice Nicoleta Drăgoescu, Andreea Stănculescu, Marius Papurică, Claudiu Rafael Barsac, Adelina Baloi, Ovidiu-Horea Bedreag

**Affiliations:** 1 Department of Anesthesia and Intensive Care, Faculty of Medicine, Titu Maiorescu University, Bucharest, Romania; 2 Department of Anesthesia and Intensive Care, CF2 Clinical Hospital, Bucharest, Romania; 3 Department of Anesthesia and Intensive Care, Victor Babeş University of Medicine and Pharmacy, Timişoara, Romania; 4 Pius Brânzeu Clinical County Hospital, Timişoara, Romania; 5 Faculty of Medicine, “Carol Davila” University of Medicine and Pharmacy, Bucharest, Romania; 6 Department of Cardiovascular Surgery, Clinical Emergency Hospital Bucharest, Bucharest, Romania; 7 Department of Anesthesiology and Intensive Care, Faculty of Medicine, University of Medicine and Pharmacy of Craiova, Craiova, Romania

**Keywords:** equipment quality AICU, healthcare management, infrastructure AICU, intensive care, quality in anesthesia and intensive care units, safety culture hospital

## Abstract

**Background:**

Anesthesia and Intensive Care Units (AICUs), hereafter referred to as Intensive Care Units (ICUs), represent high-risk clinical environments requiring advanced technology, adequate infrastructure, and specialized staff. This study explored healthcare professionals' perceptions of these structural factors and their influence on service quality across public and private hospitals in Romania.

**Methods:**

A cross-sectional quantitative survey was conducted between 4 and 28 November 2024, involving 109 respondents (52.3% nurses, 22.9% ICU specialists, 17.4% residents, and 5.5% senior consultants). Data were collected using a 20-item online questionnaire distributed via institutional and professional networks using a convenience sampling strategy.

**Results:**

Results indicated that both sectors perceived their technological infrastructure as generally adequate, with slightly higher ratings in private institutions; however, differences were small and not statistically significant (χ^2^ = 3.03, df = 4, *p* = 0.553, Cramér's V = 0.17). The most frequent challenges reported were staff shortages and uneven protocol implementation. Moderate correlations were observed between equipment quality, safety perception, and staff motivation. Both groups identified investment in equipment, continuous training, and digitalisation as top priorities for improvement. No statistically significant differences were observed between public and private hospitals for most variables (*p* > 0.05).

**Conclusions:**

The findings reflect healthcare professionals' perceptions of structural and organizational factors in ICUs. Equipment availability, infrastructure, and staffing were identified as important perceived elements associated with service quality. Given the cross-sectional design and perception-based data, results should be interpreted cautiously, particularly given the limited sample size and perception-based design. No statistically significant differences were observed between public and private hospitals for most variables (*p* > 0.05).

## 1 Introduction

Intensive Care Units (ICUs) are complex clinical environments requiring specialized staff, appropriate infrastructure, and advanced medical technologies. The structural components of high-risk clinical settings including intensive care units establish patient safety standards which affect physician choices and medical treatment delivery. The international health policy frameworks state that healthcare services need basic structural elements which include safe medical equipment and operational facilities and supporting organizational systems to achieve better quality and minimize preventable medical errors (1, 2).

Despite extensive literature describing ICU challenges such as staffing shortages and infrastructure limitations, fewer studies have explored how healthcare professionals perceive the interaction between structural resources and organizational factors in shaping service quality, particularly in Eastern European healthcare systems.

In Romania, data on perception-based evaluation of ICU structural and organizational determinants remain limited.

ICUs face sustainability challenges due to workforce deficits, aging infrastructure, and uneven funding (3, 4).

European healthcare systems have faced persistent challenges related to workforce shortages and variability in infrastructure, particularly in intensive care settings (3). The combination of high workload, resource scarcity, and limited access to updated technologies has brought renewed attention to the need for strategic investment in critical care infrastructure. Prior research emphasizes that sustained investments in technology and maintenance are strongly associated with improved safety culture and reduced incidence of adverse events (4).

This study aims to assess healthcare professionals' perceptions of structural and organizational factors in ICUs and their association with perceived service quality. In addition, it seeks to address the following research question: “Which factors are perceived by healthcare professionals as the major determinants of quality in Intensive Care Units?”.

Based on these premises, the study tests the following hypotheses:

H1: Perceived adequacy of equipment and infrastructure is associated with perceived service quality.

H2: Staff-related challenges and protocol-related issues are frequently reported in ICU settings.

Through this analysis, the study contributes to a better understanding of how resource-related factors shape perceptions of quality and safety in intensive care, providing empirical insights to provide context-specific insight into the Romanian ICU setting.

## 2 Materials and methods

### 2.1 . Study design and participants

This was a cross-sectional observational study conducted between 4 and 28 November 2024.

**Figure 1:**
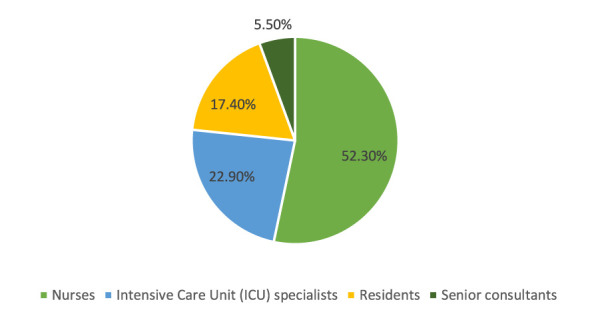
Distribution of participants and workplace setting.

Health professionals from ICUs all over Romania were invited to participate to this survey. Since the study is an observational study, based on a non-probabilistic convenience sampling method, participants were contacted via e-mail of national medical associations and online communities of specialists in the field of Anesthesia and Intensive Care and/or Emergency Medicine. The survey link was distributed via institutional email lists, national professional societies, and closed online groups dedicated to anesthesia and intensive care professionals. Participants accessed the questionnaire voluntarily through these channels. The survey was completed online and the questionnaire was distributed and promoted at national level. We cannot guarantee an approximately proportional representation of all regions of Romania. However, participants come from a variety of clinical settings and work positions within ICUs and other intensive care departments from public and private hospitals and university affiliated or independent medical units. Due to the sampling method used, public (59.6%) vs. private (40.4%) hospital settings are over-represented in this study. However, due to the nature of the study as a voluntary online survey and the recruitment strategy used, the study may be subject to self-selection bias whereby a self-selected sample of professionals predominantly active in online professional networks and/or interested in quality is recruited for the study. Missing data were handled by excluding incomplete questionnaires. No imputation methods were applied. The research team cleaned the data by removing duplicate and incomplete survey responses before starting their analysis of the 109 valid questionnaires. The research participants consisted of 52.3% nurses and 22.9% ICU specialists and 17.4% residents and 5.5% senior consultants (Figure 1).

In terms of institutional affiliation, 59.6% of respondents were employed in public hospitals, while 40.4% worked in private healthcare institutions. This distribution reflects the composition of respondents accessible through the recruitment strategy and allows exploratory comparison between organizational contexts.

### 2.2 . Data collection instrument

Data were collected using a structured online questionnaire developed through Google Forms, a practical platform for healthcare research that ensures broad accessibility and rapid data capture (5). The instrument was designed to assess perceptions of resource adequacy, teamwork, safety culture, and organizational support in ICUs. It consisted of 20 items, distributed across five thematic domains:

- Professional training and competence;

- Technological resources and infrastructure;

- Compliance with safety protocols and infection prevention;

- Communication, teamwork, and managerial support;

- Difficulties encountered and improvement measures.

The questionnaire was developed based on established theoretical frameworks in healthcare quality and patient safety, including instruments assessing ICU safety culture, organizational performance, and resource adequacy (6–17). Item selection was informed by previously published studies evaluating structural and organizational determinants of care quality in critical care settings and was adapted to the Romanian ICU context through expert consensus. Content validity was ensured through review by a panel of specialists in anesthesia and intensive care, who assessed the relevance, clarity, and clinical applicability of each item.

Responses were recorded using a five-point Likert scale ranging from 1 (“very unsatisfactory” or “to a very small extent”) to 5 (“very satisfactory” or “to a very large extent”). Likert-scale responses were treated as ordinal variables, while categorical variables included professional role and hospital type. The questionnaire also included socio-demographic and professional variables such as age, gender, professional role, years of experience, and type of healthcare institution. Internal consistency was assessed using Cronbach's alpha (α), with values ≥ 0.70 considered acceptable. Cronbach's alpha values refer to individual questionnaire domains and ranged between 0.78 and 0.89 (18).

A pilot test was conducted with 10 ICU professionals. Minor revisions were made based on feedback. Pilot participants were excluded from the final dataset.

For questions regarding difficulties, determinants of quality, and improvement measures, multiple responses were allowed.

Due to the sample size, exploratory factor analysis was not performed. Therefore, construct validity could not be formally assessed, which represents a limitation of the measurement approach.

### 2.3 . Data analysis

Data were analyzed using IBM SPSS Statistics version 26.0 (IBM Corp., Armonk, NY, USA). Descriptive statistics were used to summarize participant characteristics and main study variables. Differences between respondents working in public and private hospitals were examined using Chi-square (χ^2^) tests for categorical variables and Mann–Whitney U tests for ordinal or non-normally distributed data. Effect sizes were calculated where appropriate. Associations between key constructs, including perceived equipment quality, infrastructure adequacy, safety perception, and staff motivation, were assessed using Spearman's rank correlation coefficient (ρ). Correlation strength was interpreted as weak (ρ < 0.30), moderate (0.30 ≤ ρ < 0.50), or strong (ρ ≥ 0.50). Statistical significance was set at p < 0.05 for all analyses. To further explore independent predictors of perceived service quality and staff motivation, a multivariable regression analysis was considered. Variables showing significant or near-significant associations in univariate analyses (p < 0.10) were eligible for inclusion. However, due to the relatively small sample size and risk of model overfitting, a full multivariable model was not performed. This is acknowledged as a limitation of the analytical approach. Effect sizes were calculated for all relevant analyses (Cramér's V for Chi-square tests and rank-biserial correlation for Mann–Whitney U tests). Where applicable, 95% confidence intervals (CI) were reported to improve interpretability of the findings. Data distribution was assessed using the Shapiro–Wilk test, which indicated non-normal distribution; therefore, non-parametric tests were applied.

### 2.4 . Ethical considerations

The research followed the ethical standards outlined in the Declaration of Helsinki (2013 revision) and the ethical regulations governing human subject research in Romania. The study was approved by the Ethics Committee of CF2 Clinical Hospital protocol code 11 and date of approval 28 November 2023. Participation in the survey was voluntary and anonymous. All participants were informed about the purpose of the study, and completion of the questionnaire was considered as implied informed consent.

### 2.5 Limitations

This study has several limitations that should be considered when interpreting the findings. Among several limitations of the study is the study design using a non-probabilistic convenience sampling strategy, and thus the sample cannot be considered a representative sample of the total ICU workforce in Romanian hospitals. Furthermore, the recruitment strategy (on-line), through forums and some professional groups, may have introduced self-selection bias, and individuals most involved in their profession and/or individuals with a special interest for quality in patient care are more likely to have participated in the present study. Although the survey includes both public and private sector respondents from a variety of professional backgrounds, the survey does not include any geographical stratification and therefore it is not possible to assess the survey's representativeness at regional level. Our study's reliance on self-reported perceptions of ICU performance rather than objective indicators of performance also limits generalizability because these perceptions may be subject to reporting bias. What the items did group well on, however, was by section and internal consistency. The analysis was primarily based on univariate associations; multivariable modeling to identify independent predictors of service quality and staff motivation was not undertaken. The cross-sectional study design of this study does not allow any conclusions on a potential causality and only associative relationships can be proposed.

## 3 Results

### 3.1 . Perceived quality of equipment and technologies

As shown in Table 1, most respondents rated the quality of medical equipment and technologies in their units as good or very good. Specifically, 43.1% of participants from public hospitals and 36.3% from private hospitals evaluated the equipment quality as “good”, while 32.3% (public) and 34.0% (private) rated it as “very good”. Only a small proportion (6.2% public; 2.2% private) expressed dissatisfaction.

**Table 1:** Evaluation of equipment and technology quality—public vs. private hospitals.

Response category	Public (*n* = 65)	Public (%)	Private (*n* = 44)	Private (%)	Total (*N* = 109)
Very good	21	32.3	15	34.0	36
Good	28	43.1	16	36.3	44
Satisfactory	8	12.3	10	22.7	18
Neutral	4	6.2	2	4.5	6
Unsatisfactory	4	6.2	1	2.2	5
Total	65	100	44	100	109

The Chi-square (χ^2^) test was applied to examine differences between hospital types. Differences were small and not statistically significant (χ^2^ = 3.03, df = 4, p = 0.553), with a small effect size (Cramér's V = 0.17).

### 3.2 . Availability of essential equipment

Detailed results regarding the availability of essential equipment are presented in Supplementary Table S1.

### 3.3 . Perceived infrastructure and technical resources

Perceived adequacy of infrastructure is summarized in Supplementary Table S2.

### 3.4 . Major difficulties reported

When asked about major difficulties encountered in ICU work (Table 2), the most frequent response was staff shortages (55.4% in public hospitals, 50.0% in private). Other issues included inadequate protocols (30.8%) and insufficient technical resources (9.2%).

**Table 2:** Main difficulties encountered in ICU—public vs. private hospitals.

Difficulty	Public (*n* = 65)	Public (%)	Private (*n* = 44)	Private (%)	Total (*N* = 109)
Staff shortage	36	55.4	22	50.0	58
Inadequate protocols	20	30.8	13	29.5	33
Insufficient technical resources	6	9.2	4	9.1	10
Other/none specified	3	4.6	5	11.4	8

Multiple responses were allowed; percentages represent the proportion of respondents selecting each option and may exceed 100%.

No statistically significant association was observed between hospital type and reported difficulties (χ^2^ = 1.72, df = 3, *p* = 0.42), with a small effect size (Cramér's V = 0.13).

### 3.5 . Priority measures for quality improvement

A detailed distribution of proposed improvement measures is provided in Supplementary Table S3.

### 3.6 . Perceived determinants of quality in intensive care

Perceived determinants of ICU quality are presented in Supplementary Table S4.

### 3.7 . Statistical models and associations

Correlational analyses (Table 3) showed several significant relationships between technological resources and perceived quality of care.

**Table 3:** Correlations and comparative analyses between resource adequacy and quality perceptions.

Variable relationship	Statistical test	Coefficient/value	*p*-value	Interpretation
Equipment quality ↔ Satisfaction	Spearman	0.50	<0.01	Moderate positive correlation
Infrastructure adequacy ↔ safety perception	Spearman	0.47	<0.01	Moderate positive correlation
Investment frequency ↔ motivation	Spearman	0.62	<0.01	Significant positive correlation
Public vs. private (overall satisfaction)	Mann–Whitney U	1,394.5	= 0.29	No significant difference

The results revealed moderate to strong positive associations between key structural and perceptual variables.

Perceived equipment quality was moderately associated with overall staff satisfaction [ρ = 0.50, *p* < 0.01, 95% CI (0.34–0.63)], while infrastructure adequacy was associated with safety perception [ρ = 0.47, *p* < 0.01, 95% CI (0.30–0.60)].

A stronger association was observed between perceived investment in resources and staff motivation [ρ = 0.62, *p* < 0.01, 95% CI (0.48–0.73)].

No statistically significant difference was observed in overall satisfaction between public and private hospitals (U = 1,394.5, *p* = 0.29, *r* = 0.13).

These findings reflect associations between variables and should not be interpreted as evidence of independent predictors or causal relationships. A multivariable model would be required to assess independent effects. However, this was not performed due to sample size limitations.

## 4 Discussion

This study explores healthcare professionals' perceptions of the impact of structural and organizational factors upon quality of healthcare service delivered to patients and their families in the ICU setting. The findings of this study should be interpreted as perception-based and do not represent objective measures of ICU performance or patient outcomes. Findings indicate that whilst professionals consider the availability and quality of equipment and the physical and technical health environment, as well as staffing and skills mix to be important factors in the clinical environment, these are perceived as important factors associated with quality of care, based on healthcare professionals' subjective evaluations. Perceptions of having adequate equipment and facilities have been found to be associated with safety climate and nurse satisfaction in the ICU setting (5, 16–18). The research results confirm that structural adequacy is perceived as an important component associated with maintaining quality standards in ICU settings.

Although statistically significant associations were identified, the absence of multivariable modeling limits the ability to determine which factors independently influence perceived service quality or staff motivation.

Therefore, the findings should be interpreted as exploratory and perception-based, rather than representative of the entire Romanian ICU system.

Although numerical differences were observed, there were not statistically significant and should not be interpreted as meaningful differences. The absence of statistically significant differences should not be interpreted as evidence of equivalence between hospital types.

The organizations pursue identical goals which suggests that technological readiness is perceived as an important factor influencing clinical performance (19, 20). The participants explained that technology alone is not sufficient to ensure high-quality care, according to existing literature. Healthcare providers needed to practice safe clinical care through continuous education of their professional skills and by following established protocols according to (21, 22).

A positive association was observed between perceived investment in resources and staff motivation; however, this relationship should be interpreted as correlational rather than causal (23, 24). Research shows that digital systems which enable record interoperability through automated alert systems and data monitoring platforms have been suggested to support improvements in both situational awareness and preventable errors in intensive care units (25, 26). These tools achieve their best results when organizations use them as part of established systems which support both their operations and their workforce (27, 28).

**Figure 2:**
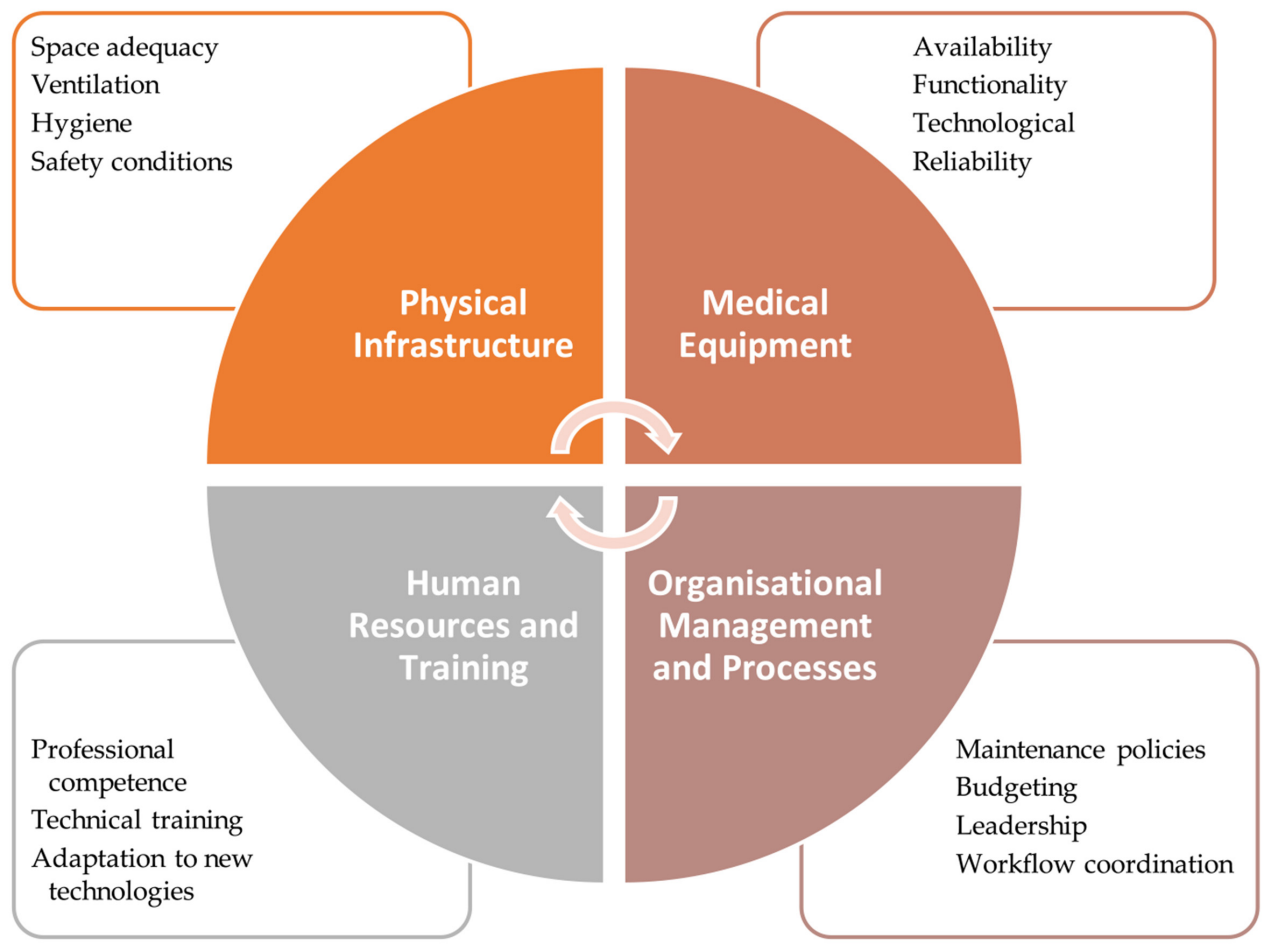
Conceptual matrix—quality of Care in anesthesia and intensive care units (ICUs).

The conceptual framework (Figure 2) illustrates the perceived interaction between structural and organizational factors in ICU settings. The research demonstrates that product quality and safety assessment by consumers depends on three essential elements which include structural components and technological frameworks and human factors.

**Figure 3:**
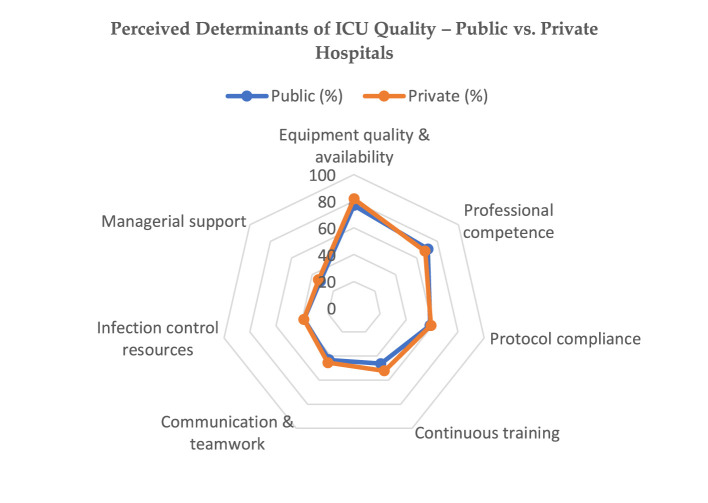
Perceived determinants of ICU quality—public vs. private hospitals.

The comparative visualization presented in Figure 3 further supports this interpretation by showing that technological infrastructure and professional competence are consistently perceived as the most influential determinants of ICU quality across both public and private hospitals. Organizational and managerial factors, while still relevant, were assigned comparatively lower priority, suggesting potential areas for future development in quality management strategies.

This study provides a perception-based analysis of the structural and organizational determinants that shape the work environment of the ICU. The findings from our study are comparable to other studies conducted in European ICUs regarding the safety culture and determinants of quality-of-care structural factors. Perceptions of availability of required equipment, staffing levels and organizational support are often reported in literature in relation to safety climate and job satisfaction in critical care settings (16–18). Our study adds general European findings to the discussion and contributes to understanding perception-based evaluation of ICU structural factors in the Romanian healthcare context.

Future studies should include formal validation of measurement instruments, including factor analysis, to strengthen the reliability and generalizability of findings.

## 5 Conclusions

This study provides insight into healthcare professionals' perceptions of structural and organizational factors in Intensive Care Units within the Romanian healthcare context. Equipment availability, infrastructure, and staffing were identified as important perceived elements associated with service quality.

Given the perception-based nature of the data and the cross-sectional design, the findings should be interpreted cautiously and do not establish causal relationships. The study contributes to the limited literature on perception-based evaluation of ICU structural factors in Eastern European healthcare systems.

## Data Availability

The raw data supporting the conclusions of this article will be made available by the authors, without undue reservation.
