# Supplementary material for: Healthcare professionals' perception of equipment and infrastructure as determinants of quality in anesthesia and intensive care units
Source: Front Med (Lausanne). 2026 May 26;13:1797446. doi: 10.3389/fmed.2026.1797446 (PMC13246601; doi:10.3389/fmed.2026.1797446)
Supplement: Supplementary file 1 [file Table_1.docx]

Supplementary Material

Healthcare Professionals’ Perception of Equipment and Infrastructure as Determinants of Quality in Anaesthesia and Intensive Care Units

Gabriel-Petre Gorecki^1,2^, Dorel Săndesc^3,4^, Horațiu Moldovan^5,6^, Alice-Nicoleta Drăgoescu^7,*^, Andreea Stănculescu^7^ , Marius Papurică^3,4,*^, Claudiu Rafael Barsac^3^, Adelina Baloi^3^, Ovidiu-Horea Bedreag^3,4^

**Supplementary Table S1. Availability of Essential Equipment**

| Response Category | Public (n = 65) | Public (%) | Private (n = 44) | Private (%) | Total |
| --- | --- | --- | --- | --- | --- |
| Very great extent | 15 | 23.1 | 11 | 25.0 | 26 |
| Great extent | 28 | 43.1 | 20 | 45.4 | 48 |
| Small extent | 11 | 16.9 | 7 | 15.9 | 18 |
| Neutral | 11 | 16.9 | 6 | 13.6 | 17 |
| Total | 65 | 100 | 44 | 100 | 109 |

**Supplementary Table S2. Infrastructure Adequacy**

| **Response Category** | **Public (n = 65)** | **Public (%)** | **Private (n = 44)** | **Private (%)** | **Total** |
| --- | --- | --- | --- | --- | --- |
| Very great extent | 16 | 24.6 | 9 | 20.4 | 25 |
| Great extent | 27 | 41.5 | 21 | 47.7 | 48 |
| Small extent | 10 | 15.4 | 7 | 15.9 | 17 |
| Very small extent | 1 | 1.5 | 0 | 0.0 | 1 |
| Neutral | 11 | 16.9 | 7 | 15.9 | 18 |
| Total | 65 | 100 | 44 | 100 | 109 |

**Supplementary Table S3. Proposed Improvement Measures**

| **Proposed Measure** | **Public (%)** | **Private (%)** | **Total (%)** |
| --- | --- | --- | --- |
| Investment in equipment/infrastructure | 81.5 | 81.8 | 81.7 |
| Continuous professional training | 64.6 | 70.5 | 67.0 |
| Stricter protocol implementation | 53.8 | 56.8 | 55.0 |
| Better communication between teams | 44.6 | 47.7 | 46.0 |
| Feedback system without sanctions | 33.8 | 38.6 | 36.0 |

*Multiple responses were allowed; percentages represent the proportion of respondents selecting each option and may exceed 100%.*

**Supplementary Table S4. Perceived Determinants of ICU Quality**

| Determinant Factor | Public (%) | Private (%) | Total (%) |
| --- | --- | --- | --- |
| Equipment quality & availability | 76.9 | 81.8 | 78.9 |
| Professional competence | 70.8 | 68.2 | 69.7 |
| Protocol compliance | 58.5 | 59.1 | 58.8 |
| Continuous training | 46.2 | 52.3 | 49.0 |
| Communication & teamwork | 43.1 | 45.4 | 44.0 |
| Infection control resources | 38.5 | 38.6 | 38.5 |
| Managerial support | 32.3 | 34.1 | 33.0 |

*Multiple responses were allowed; percentages represent the proportion of respondents selecting each option and may exceed 100%.*
